# Pre‐existing humoral immune comebacks control the development of the severe form of coronavirus disease 2019 in Gaucher patients

**DOI:** 10.1002/ctd2.96

**Published:** 2022-06-29

**Authors:** Manoj Kumar Pandey

**Affiliations:** ^1^ Division of Human Genetics Cincinnati Children's Hospital Medical Center Cincinnati Ohio USA; ^2^ Department of Pediatrics University of Cincinnati College of Medicine Cincinnati Ohio USA

**Keywords:** autoantigens, corona virus, immunoglobulins, infection, rare genetic disease

## Abstract

Coronavirus disease 2019 (COVID‐19) and the Gaucher disease (GD) exhibit lot of resemblances in induction of innate and adaptive immune inflammation that include the immune cells activation and the massive generation of pro‐inflammatory cytokines, chemokines, and growth factors, which are all critical for propagation of the disease process and the multiple organ damage. However, majority of the GD patients have not revealed the expansion of severe form of the COVID‐19. This study suggests that the pre‐existing humoral immunity influence the devlopment of strong network of antibodies to different structural proteins of SARS‐CoV2 in GD patients with COVID‐19. Such antibodies and virus proteins interaction cause the comprehensive neutralization of SARS‐CoV2 and provides protection from the development of severe form of COVID‐19 in GD patients. This information could be helpful for better understanding of the disease mechanism as well as the development of additional potential therapy that could stop the growth of the severe symptoms and/or death in GD patients with COVID‐19.

## 1 GAUCHER DISEASE

Gaucher disease (GD) affects approximately 1/40 000–1/60 000 live births and happens due to mutations in *GBA1 (human)/Gba1(mice)* that lead to the functional disruption of the encoded lysosomal enzyme, acid β‐glucosidase and the consequent excess tissue accumulation of glucosylceramide (GC).^1^ Several of the immune cells, that is, macrophages (Mɸs), dendritic cells (DCs), T, and B cells and their effector pro‐inflammatory cytokines, that is, interferon gamma (IFNγ), tumour necrosis factor alfa (TNFα), interleukin 1α [IL1α], IL1 beta [IL1β], IL6, IL12, IL17, IL18, IL21 and IL23, chemokine C‐C motif ligand; CCL, (e.g. CCL1, CCL2, CCL3, CCL4, CCL5, CCL6, CCL9, CCL17, CCL18 and CCL22), chemokine C‐X‐C motif ligand; CXCLs, (e.g. CXCL1, CXCL2, CXCL8, CXCL9, CXCL10, CXCL11, CXCL12 and CXCL13), and growth factors, that is, transforming growth factor‐β1, hepatocyte growth factor, Mɸ colony‐stimulating factor (MCSF), granulocyte colony‐stimulating factor (GCSF), and granulocyte Mɸ colony‐stimulating factor (GMCSF) and their impact in the development of anaemia, thrombocytopenia, hypergammaglobulinemia, splenomegaly, hepatomegaly, bone and brain defects have been observed in GD.^1^

### 1.1 Coronavirus disease

The coronavirus disease 2019 (COVID‐19) is caused by severe acute respiratory syndrome coronavirus 2 (SARS‐CoV2) that infected around 534 million individuals and is responsible for around 6.31 million deaths worldwide. SARS‐CoV2 genome encodes four major proteins, that is, spike (S; essential for the entry into host cells), envelope (E; responsible for viral membrane curvature and binding to the nucleocapsid), membrane (M; bind to the viral RNA genome and ensure the maintenance of the RNA in a ‘beads‐on‐a‐string’ conformation), and nucleocapsid (N; needed for viral replication and disease pathogenesis), which are critical for massive generation of pro‐inflammatory cytokines, (e.g. IFNγ, TNFα, IL1, IL2, IL5, IL6, IL7, IL8, IL9, IL10, IL12 and IL17), CCL chemokines, (e.g., CCL2, CCL3 and CCL5), CXCL chemokines, (e.g., CXCL9, CXCL10, and CXCL11), and growth factors, that is, TGFβ, GCSF, GMCSF, vascular endothelial cell growth factor, fibroblast growth factor and platelet‐derived growth factor, which are all critical for development of symptoms, (e.g. dry cough, taste and smell loss, fatigue, malaise, high fever, difficulty breathing, acute respiratory distress syndrome, pneumonia, respiratory failure and death) in COVID‐19 patients.^2^

### 1.2 SARS‐CoV 2 infected GD patients and the status of COVID‐19

High‐level mortality of COVID‐19 was observed in older age group individuals (>60 years of age) and people with immunocompromised and morbid conditions, that is, sepsis, acute cardiac injury, heart failure, and multi‐organ, (e.g. liver, spleen, kidney and brain) defects.^3^ It is, therefore, speculated that GD patients could be at high risk for developing severe COVID‐19. Strikingly, most of the SARS‐CoV‐2 infected GD patients have shown mild symptoms and survived.^4^ Only one GD patient (a 79‐year‐old man experiencing Alzheimer's dementia and prior renal malignancy) has died due to SARS‐CoV2 infection.^5^ The exact mechanism by which SARS‐CoV2‐infection has been unsuccessful to develop severe COVID‐19 in the majority of the GD patients is not known.

### 1.3 The promising mechanism of SARS‐CoV 2 neutralization in GD patients with COVID‐19

B cells are classified into B1 and B2 cells, where the former cells show immunoglobulin M (IgM)^hi^ IgD^lo^, and B220^+^ CD11b^+^ surface expression as compared to the later cells. B‐1 cells mainly reside in body cavities such as the pleura and peritoneum whereas B‐2 cells are mainly located in secondary lymphoid organs such as the spleen and lymph nodes.^6^ Both cell types can also be found in lower frequencies in the bone marrow.^6^ The B1 cell secrete natural IgM antibodies to self‐antigens, that is, phosphatidylcholine, single‐stranded deoxyribonucleic acid, ribonucleoprotein, cell surface Thy‐1 antigen, and the rheumatoid factor. In contrast, B2 cells (conventional B cells) express intermediate levels of IgM and IgD, differentiate into memory and plasma cells and undergo immunoglobulin isotype switching.^6^ B‐1 cells and its function in development of autoantigen, (e.g. cardiolipin, β_2_ glycoprotein‐I, pyruvate dehydrogenase, deoxyribonucleic acid, ribonuclear protein, nuclear, neutrophil cytoplasmic, sulfatide and rheumatoid factors)‐ specific IgA, IgM, and IgG antibodies have been described in GD patients with N370S and L444P mutations.^6^ Increased frequencies of certain B cell subsets including B220^+^ CD19^+^, B220^+^CD138^+^, B220^+^CD19^+^Fas^+^GL7, CD27^hi^ CD38^hi^ or CD45^lo^CD19^–^CD38^+^CD138^+^ cells that are associated with increased production of GC‐specific IgG2a/c and IgG2b autoantibodies in *Gba1^9V/–^* mouse model of GD and high levels of GC‐specific IgG1 autoantibodies and lower levels of IgG2 and IgG3 autoantibodies have been observed in GD patients.^1^ These GC‐specific IgG autoantibodies in Gba1^9V/–^ mice and in GD patients formed GC‐specific IgG immune complexes (GC‐ICs).^1^ Such IgG‐ICs trigger their effector function when they crosslink with activating FcγRs, for example, FcγRI, FcγRIII, and FcγRIV in mice and FcγRI, FcγRIIA, FcγRIIC and FcγRIIIA in humans or the inhibitory FcγRIIB in mice and humans.^6^

**FIGURE 1:**
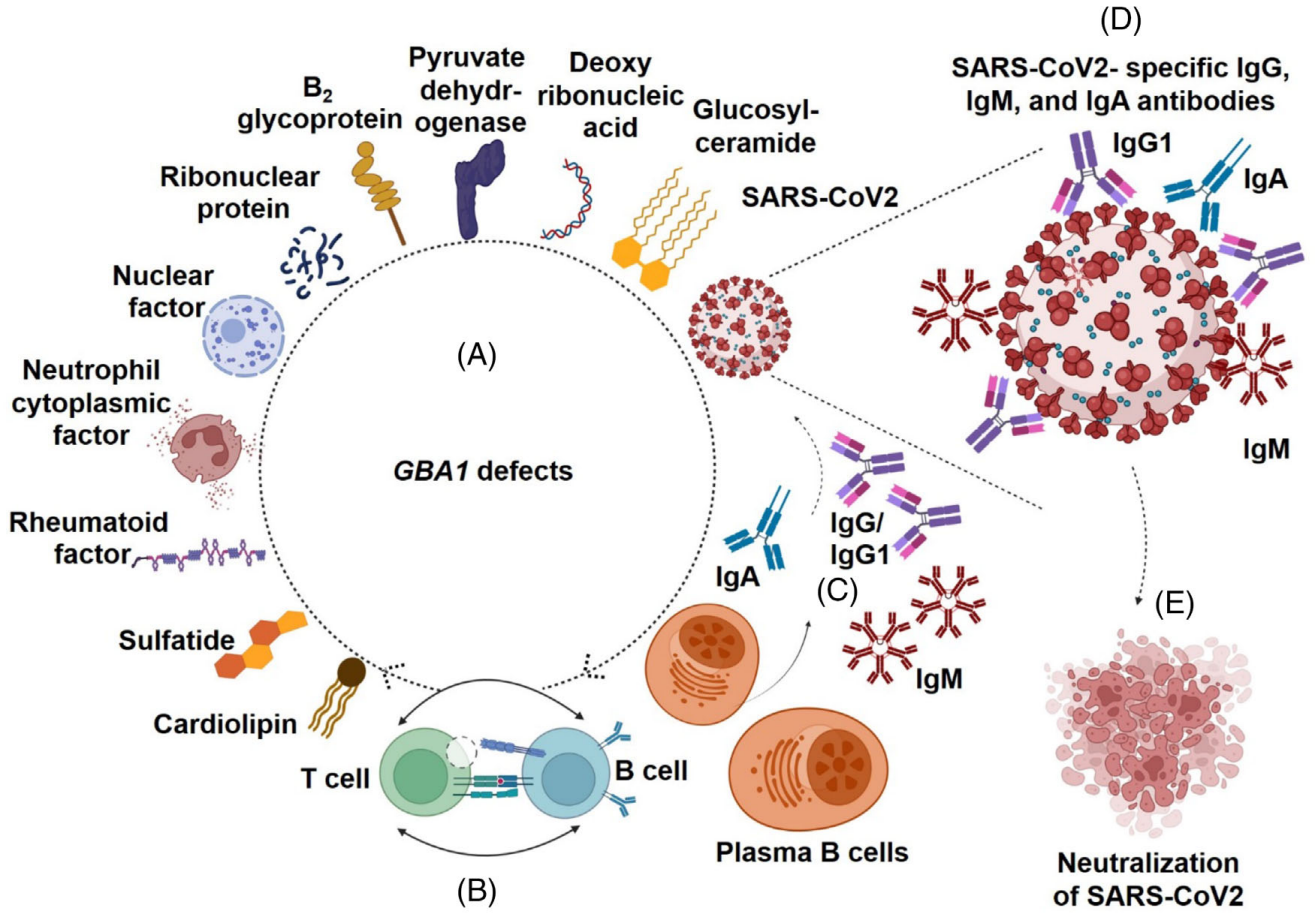
**The Immune reactions against the SARS‐CoV2 in Gaucher Patients with COVID‐19**. GBA1 defects lead to the excess development and release of several autoantigens, (e.g., glucosylceramide, cardiolipin, β_2_ glycoprotein‐I, pyruvate dehydrogenase, deoxyribonucleic acid, ribonuclear protein as well as the nuclear, neutrophil cytoplasmic, sulfatide, and rheumatoid factors) in Gaucher Patients (A). B cells mediated excess uptake, processing, and the presentation of such antigens to the specific T cells lead to the differentiation of B cells into the plasma B cells and the increased production of IgG, IgM, and IgA autoantibodies specific to the indicated antigens (B‐C). It is therefore most likely that upon the SARS‐CoV2 attack, these pre‐existing humoral immune responses facilitate and guide in quick and strong development of SARS‐CoV2's proteins, (e.g., S, RBD, and N)‐ specific IgG1, IgM, and IgA antibodies and this situation eventually caused the neutralization of the SARS‐CoV2 and the protection from the development of severe and chronic form of COVID‐19 in GD patients (D‐E).

The SARS‐CoV‐2 infection has shown the sustained development of memory and long lived bone marrow plasma B cells and the production of IgG, IgM, and IgA antibodies to S, RBD of S, and N proteins.^7^, ^8^, ^9^ Analysis of antibody Fc regions revealed that IgG1 binding to activating FcγR receptors contributes to optimal protection against SARS‐CoV2.^10^ However, the majority of the COVID‐19 patients have not shown complete protection from reinfection due to short term or no development of SARS‐CoV2‐specific antibodies.^7^, ^8^, ^9^ Elevated levels of GC‐specific IgG1 antibodies and activating FcγR have been observed in GD.^1^ SARS‐CoV2 infected GD patients have shown the development of IgG antibodies to S protein of SARS‐CoV2.^4^ More studies are needed to detect and characterize the exact nature of IgM, IgA, and IgG subtypes, (e.g. IgG1, IgG2, IgG3 and IgG4) antibodies to S, RBD of S, and N proteins of SARS‐CoV2 in GD patients with COVID‐19. However, based on the findings of this paper, it is speculated that the activation of humoral immune responses and the resultant development of IgG1, IgM, and IgA antibodies to auto‐antigens in GD patients act as a strong marketer for continuous development of IgG1, IgM, and IgA antibodies to different structural proteins, (e.g. S, RBD of S and N) of SARS‐CoV2 in GD patients with COVID‐19. Such vigorous and guided immunological setting caused the comprehensive trapping and neutralization of SARS‐CoV2 and the resultant protection from the development of severe stage of COVID‐19 in GD patients (Figure 1A–E). This detail could be helpful for better understanding of the disease mechanism and better cure of GD patients with COVID‐19 and/or those showing poor response to existing vaccines.

### CONFLICT OF INTEREST

Author affirms that this manuscript was completed in absence of any commercial or financial interactions that could be interpreted as a potential conflict of interest.

### AUTHOR CONTRIBUTIONS

MKP has intellectualized and transcribed the study, led the literature search, did the proofreading, and approval of final version of the manuscript.
